# Bi-Level Positive Airway Pressure for Non-invasive Respiratory Support of Foals

**DOI:** 10.3389/fvets.2021.741720

**Published:** 2021-09-29

**Authors:** Sharanne L. Raidal, Chee Sum Melanie Catanchin, Lexi Burgmeestre, Chris T. Quinn

**Affiliations:** School of Animal and Veterinary Sciences, Charles Sturt University, Wagga Wagga, NSW, Australia

**Keywords:** non-invasive ventilation (NIV), equine critical care, neonatology, equine respiratory physiology, respiratory insufficiency

## Abstract

Respiratory insufficiency and pulmonary health are important considerations in equine neonatal care. As the majority of foals are bred for athletic pursuits, strategies for respiratory support of compromised foals are of particular importance. The administration of supplementary oxygen is readily implemented in equine practice settings, but does not address respiratory insufficiency due to inadequate ventilation and is no longer considered optimal care for hypoxia in critical care settings. Non-invasive ventilatory strategies including continuous or bi-level positive airway pressure are effective in human and veterinary studies, and may offer improved respiratory support in equine clinical practice. The current study was conducted to investigate the use of a commercial bi-level positive airway pressure (BiPAP) ventilator, designed for home care of people with obstructive respiratory conditions, for respiratory support of healthy foals with pharmacologically induced respiratory insufficiency. A two sequence (administration of supplementary oxygen with, or without, BiPAP), two phase, cross-over experimental design was used in a prospective study with six foals. Gas exchange and mechanics of breathing (increased tidal volume, decreased respiratory rate and increased peak inspiratory flow) were improved during BiPAP relative to administration of supplementary oxygen alone or prior studies using continuous positive airway pressure, but modest hypercapnia was observed. Clinical observations, pulse oximetry and monitoring of expired carbon dioxide was of limited benefit in identification of foals responding inappropriately to BiPAP, and improved methods to assess and monitor respiratory function are required in foals.

## Introduction

Respiratory disease has long been recognized as of considerable economic importance in newborn foals (1), and as an important cause of morbidity and death in neonates presented for veterinary care (2, 3). Optimal respiratory support is highly desirable to optimize survival and preserve respiratory function in animals bred largely for their athletic potential.

The use of non-invasive ventilation (NIV) is now widely regarded as the most effective approach for respiratory support of human neonates (4, 5), with continuous positive airway pressure (CPAP) shown to reduce the number of preterm infants requiring admission to neonatal intensive care (6), and to decrease the risk of bronchopulmonary dysplasia or death in neonates requiring respiratory support (7). The technique involves the delivery of a constant positive (greater than atmospheric) pressure to the airway and preserves spontaneous respiration. The physiological effects are complex and likely to vary depending on the underlying pathology (8), but benefits have been attributed to increased functional residual capacity, decreased work of breathing and reduced airway resistance (4). Previous studies have demonstrated that CPAP is associated with improved respiratory function in a number of veterinary species (9–12). CPAP has recently been shown to improve gas exchange in healthy foals with pharmacologically induced respiratory suppression (13), however hypercapnia was observed in treated foals in this study, and has been observed previously in anesthetized horses during CPAP (10, 11, 14).

Bi-level positive airway pressure (BiPAP) is also recognized for the management of respiratory insufficiency in human neonates, and has demonstrated improved treatment outcomes in preterm human neonates in comparison to CPAP (15, 16). By using lower expiratory pressures, BiPAP promises improved expiratory function and is recommended for management of conditions associated with hypercapnia, such as chronic obstructive airway disease or asthma (17–19). In human patients with obstructive airway conditions, expiratory airflow limitations may cause increased PaCO_2_ due to overdistension of alveoli and consequent increased alveolar dead space (20–23), an effect which has been termed dynamic hyperinflation (24).

Whereas, human neonates exhale passively (25), both inspiration and expiration are active processes, requiring muscular effort, in foals (26). Foals might therefore be predisposed to expiratory flow limitations and retention of CO_2_ if active breathing strategies are unable to overcome expiratory pressures during CPAP, and the technique might be thus be associated increased intrinsic positive end-expiratory pressure (PEEPi) and alveolar overdistension, and hence predispose to hypercapnia. Lower expiratory pressures during BiPAP might be expected to facilitate expiration and ameliorate this effect. The current study was undertaken to determine whether a commercially available bi-level respiratory device, available for the home care of people with respiratory disease, might represent a ventilatory support strategy in healthy foals with pharmacologically induced respiratory insufficiency. We hypothesized that BiPAP would be associated with improved respiratory function, and with less CO_2_ retention, than observed previously during CPAP (13). Intentionally, the study was designed to evaluate low cost intervention and monitoring strategies that might be safely implemented in equine practice or on farm.

## Materials and Methods

### Animals

Six healthy foals (two colts, four fillies) of mean age 47.7 days (range 44–52 days) and mean body weight 111.2 kg (range 86–125 kg) were available for the current study. All foals were Connemara cross breeding and were normal on veterinary examination at the time of recruitment into the study, and prior to each intervention.

### Experimental Design

A randomized crossover design was used with the first treatment assigned (either BiPAP or mask O_2_) determined by coin toss (Supplementary Table S1). Treatment order was reversed for the next data collection day for each foal. A cross-over design was selected for the interventional study to further increase statistical power and to control for individual differences and possible effects attributable to treatment order. The interval between intervention periods ranged from 3 to 6 days, and the cross over design included treatment in both left (Phase 1) and right lateral recumbency (Phase 2) for each foal. The study protocol was approved by the Charles Sturt University Animal Care and Ethics Committee (ACEC A18044).

### Sedation and Sampling Protocol

Prior to each study, foals were manually restrained for veterinary examination and collection of baseline arterial blood samples from the carotid artery (T-1). Briefly, the right carotid artery was palpated in the distal cervical region; the skin was swabbed with 70% ethanol, and the jugular vein proximal to the injection site was occluded. A 23 G, 32 mm needle was introduced into the carotid artery at a 45° angle and blood collected directly into a pre-heparinised syringe (BD Heparinised Syringes, Becton Dickinson, North Ryde, Australia). Pressure was applied to the arteriopuncture site for 1–2 min. A 16 G, 89 mm catheter (Terumo Surflo, Macquarie Park, Australia) was aseptically placed in the jugular vein of each foal and a baseline sample of venous blood was collected directly into pre-heparinised syringes from the catheter for each foal prior to sedation. Blood gas analysis (GEM Premier, Model 3500; Abacus ALS, Macquarie Park, Australia) was performed on anaerobically collected samples to determine partial pressures of oxygen and carbon dioxide (PaO_2_ and PaCO_2_, respectively), hemoglobin saturation (sO_2_) and pH. Spirometry was performed on standing foals as previously described (13) by application of a large veterinary anesthesia mask (SurgiVet large canine mask, product number 32393B1; Sound Veterinary Equipment, Rowville, Australia) placed on the foal's muzzle in such a way as to exclude air leaks and to minimize dead space, but not prevent opening of the nares. A respiratory flow head (Respiratory Flow Head 300 L, MLT300L, ADInstruments, Bella Vista, Australia) and gas sampling port were connected to the anesthesia mask. Dead space of this apparatus was 60 mL (measured by water displacement). Data were collected for up to 60 s (sufficient to ensure 10 artifact free breath cycles) in unsedated foals, and analyzed using PowerLab 4/25, Gas Analyser ML206, and LabChart 8 software (ADInstruments, Bella Vista, Australia). Tidal volume (Vt), peak inspiratory and peak expiratory air flow (PIF, PEF), and the duration of inspiratory (Ti) and expiratory (Te) phases were determined by post-sampling analysis of six consecutive and artifact free breath cycles representative of tidal breathing. Spirometry, inspired and expired gas analysis (FiO_2_, FeO_2_, FiCO_2_, and FeCO_2_) were performed following calibration of the spirometer pod using a using a seven liter certified calibration syringe (Hans Rudolph Incorporated, Shawnee, Kansas, USA) and the gas analyser was calibrated using a two point calibration of room air (20.9% O_2_, 0.04% CO_2_) and Carbogen (95% O_2_, 5% CO_2_; BOC Gas, Wagga Wagga, Australia). Pulse oximetry (SpO_2_) was performed, when possible, with a transmission probe (Avant 2120, Nonin Medical Inc., Plymouth, MN, USA; distributed by Proact Medical Systems, Port Macquarie, NSW, Australia) placed on the tongue. Results were recorded when the signal was constant over 2 min, pulse rate matched heart rate, and pulse strength was satisfactory. Pulse oximetry was not possible when the anesthetic mask was in place.

Diazepam (0.2 mg/kg) was administered via the intravenous catheter, and spirometry (T0) was repeated 5 min following treatment. Fentanyl (5 μg/kg; Hospira, Melbourne, Australia) and xylazine (0.2 mg/kg; Illium Veterinary Products, Glendenning, Australia) were then administered via the jugular catheter, and foals were placed in lateral recumbency. A 22 G, 25 mm polyurethane catheter (Surflo, Terumo Australia Pty Ltd, Macquarie Park, Australia) was placed aseptically into the lateral metatarsal artery, and an arterial blood sample collected anaerobically (T0). Foals were monitored by determination of cardinal signs (HR, RR, temperature, MAP), arterial blood gases (PaO_2_, PaCO_2_, sO_2_, pH), spirometry and inspired/expired gas analysis. Spirometry data were collected over 20–40 s, after collection of arterial blood samples, to minimize any effects attributable to apparatus dead space. Respiratory suppression was then induced by continuous infusion of fentanyl (0.005 mg/kg/h) and xylazine (0.7 mg/kg/h) in 0.9% sodium chloride delivered via syringe pump (Alaris IMED Gemini PC-1 infusion pump; VetQuip Pty Ltd, Erskine Park, NSW), commencing immediately after collection of T0 samples. Samples were again collected after 10 min spontaneous respiration (10 min following commencement of the fentanyl-xylazine CRI, T1), and respiratory support (BiPAP or O_2_ supplementation) was commenced 10 min following collection of T1 samples. The treatment and sample schedule is shown in Table 1.

**Table 1:** Treatment and sampling schedule.

**Time**		**Position**	**Sedation**	**Respiratory support**	**Samples**				
					**Spiro**	**ABG**	**TPR**	**MAP**	**SpO_**2**_**
T-1	0 min	Standing	Nil	Nil	✓	✓	✓		
T0	10 min	Standing	Diazepam 0.2 mg/kg	Nil	✓		✓		
		Lateral	Fentanyl 5 μg/kg + xylazine 0.02 mg/kg; commence CRI	Nil		✓		✓	✓
T1	20 min	Lateral	CRI	Nil	✓	✓	✓	✓	✓
T2	30 min	Lateral	CRI	O_2_ or BiPAP	✓	✓	✓	✓	
T3	40 min	Lateral	CRI	Nil	✓	✓	✓	✓	✓
T4	50 min	Lateral	CRI	BiPAP or O_2_	✓	✓	✓	✓	
T5	60 min	Lateral	End CRI	Nil	✓	✓	✓	✓	✓

*Baseline data were collected from standing, unsedated foals at T-1. Spirometry was performed at T0 in standing foals 5 min following administration of diazepam. Arterial blood gas, heart rate, respiratory rate, and temperature were collected from recumbent foals within 5–10 min of the administration of a bolus injection of fentanyl and xylazine. A continuous infusion of fentanyl and xylazine in 0.9% sodium chloride delivered via syringe pump was commenced at the end of T0 (10 min lateral recumbency). Foals were randomized, in pairs, to receive oxygen administration (8 L/min, O_2_) or non-invasive ventilation (BiPAP at inspiratory pressure of 15 cmH_2_O and expiratory pressure at 5 cmH_2_O, with oxygen administration at 8 L/min) at T2, with the reciprocal treatment administered at T4. Treatment order (BiPAP/oxygen administration) was reversed in the second replicate. Spiro, spirometry and inspired/expired gas analysis; ABG, arterial blood gas; TPR, temperature, heart (pulse) rate, respiratory rate, and mean arterial pressure; SpO_2_, pulse oximetry*.

### BiPAP and O_2_ Supplementation

Respiratory support (BiPAP or O_2_ supplementation) was delivered via the large veterinary anesthesia mask used for spirometry measurements. The mask was connected to a vented non-rebreathing elbow valve (Oracle 2 Vented Non-rebreathing Valve, 400HC206, Fisher and Paykel Healthcare, Nunawading, Victoria, Australia), hence to a commercial, bi-level pressure support ventilator specifically designed for non-invasive mask ventilation (VPAPTM III ST, ResMed Ltd., Bella Vista, NSW) via standard air tubing (ResMed Ltd., Bella Vista, NSW) of two meter length (Supplementary Figure S1). Based on previous findings, a minimum respiratory rate (RR) of 15 bpm was set on the ventilator, such that a breath would be initiated if spontaneous ventilation fell below this rate. Inspiratory pressure (IPAP) was set at 15 cmH2O, and expiratory pressure (EPAP) was set at 5 cmH2O. The maximum duration of IPAP was 1.2 s to prevent prolonged inspiration, minimum duration of IPAP set at 0.5 s to prevent false triggering, and the I:E ratio was 1:2.3. Oxygen (8 L/min) was delivered into the system between the non-rebreathing valve and the ventilator tubing, as shown (Supplementary Figure S1). A pressure manometer (Advanced Anesthetic Services, Gladesville, Australia) was connected to the BiPAP ventilator to enable circuit pressure monitoring. Spirometry and gas sampling were performed by inserting the respiratory flow head and gas sampling port between the one-way valve and mask, as shown in Supplementary Figure S1, at the end of each respiratory intervention and after collection of arterial blood samples. Temperature, HR, RR, and MAP were recorded over the final 2 min of each respiratory intervention. Pulse oximetry (SpO_2_) could not be performed during mask administration of O_2_ or BiPAP because the transmission probe could not be placed on the tongue.

### Statistical Methods

Power analysis from previous studies demonstrated that a sample size of six foals would discriminate differences in PaO_2_ and PaCO_2_ of 15 and 5 mmHg, respectively, with a power > 0.80 and α = 0.05. Data were tested for normality by the Shapiro-Wilks test and explored using appropriate descriptive statistics. The effect of replicate (Phase 1 or Phase 2) and sequence (O_2_ or BiPAP at T2 with reciprocal treatment at T4) were evaluated by fitting separate mixed effects models using restricted maximum likelihood (REML) with time and replicate or sequence as random factors and foal as a fixed factor. In the absence of significant replicate or sequence effects, treatment effects (BiPAP vs. O_2_) were determined by mixed effects models with time as a random factor and subject as a fixed factor and *post-hoc* testing by Tukey's method. Non-parametric results were analyzed by Kruskal-Wallis test, with *post-hoc* testing by Dunn's method. Relationships between PaO_2_, sO_2_, and pulse oximetry (SpO_2_), and between maximum CO_2_ in expired air (CO_2_max) and PaCO_2_, were explored using Pearson's correlation; Bland-Altman analyses were used to assess agreement between these indices. Unless specifically stated, data satisfied criteria for normality and parametric tests were used. Significance was accepted as *P* < 0.05 and all analyses were performed using Graph Pad Prism 8.4.3 for Windows (GraphPad Software, San Diego, California USA, www.graphpad.com).

## Results

No effects attributable to replicate were observed for any parameter. Effects associated with sequence (BiPAP or supplementary O_2_ at T2) are shown in Supplementary Materials. Heart and respiratory rates were highest in unsedated foals, and temperature decreased significantly throughout the study period (from 38.5 to 37.8°C). Mean arterial pressure did not change associated with time or treatment (Supplementary Figure S2).

**Figure 1:**
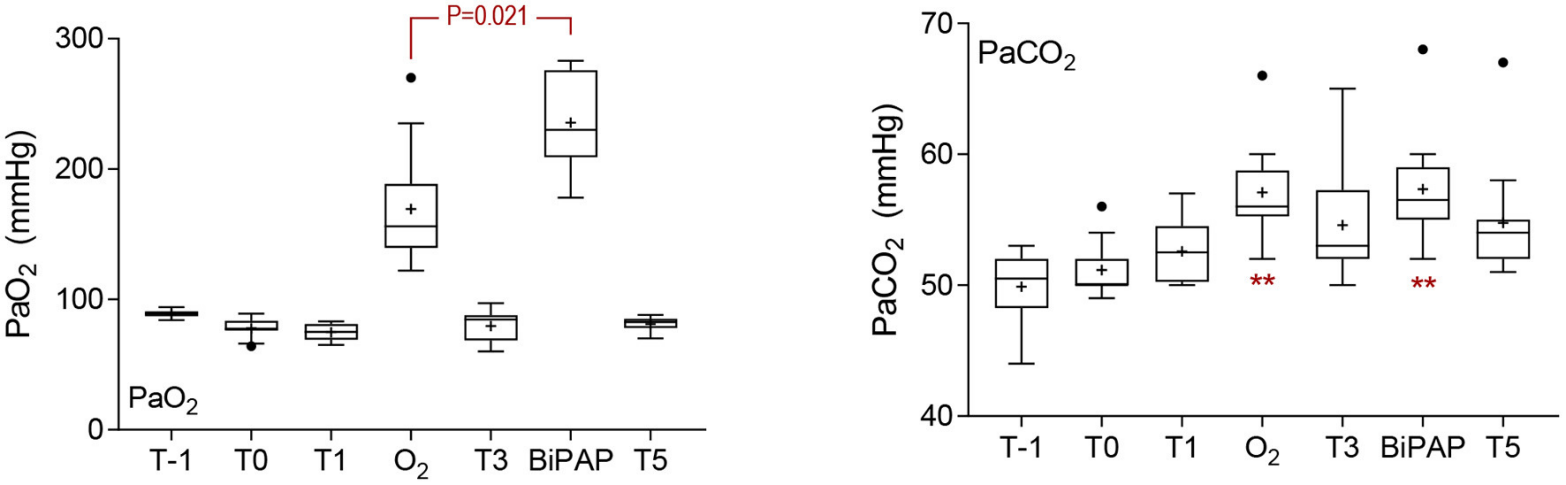
Blood gas results. Sedation was associated with a significant decrease in PaO_2_ at T0 (*P* = 0.002) and T1 (*P* = 0.004). The administration of supplementary oxygen by mask (O_2_) or during bi-level positive airway pressure ventilation (BiPAP) was associated with a significant increase in PaO_2_ (both *P* < 0.001), and results following BiPAP were significantly greater than following O_2_, as indicated. Results for arterial CO_2_ pressures were not normally distributed, and were resistant to transformation. Results following O_2_ and BiPAP were significantly greater than results at T-1 and T0, as shown (**P < 0.001), following analysis by Kruskal Wallis test. Differences in PaCO_2_ were not different following O_2_ or BiPAP (*P* = 1.000). Data are shown as mean (+), median (horizontal line), and quartiles (box), with whiskers and outliers determined by Tukey method.

Oxygenation (PaO_2_) was greater in unsedated foals (T-1) than observed in sedated foals at T0 (*P* = 0.002) or T1 (*P* = 0.004, Figure 1). The administration of supplementary oxygen alone or with BiPAP was associated with significantly increased PaO_2_ in comparison to results at all other sampling times (all *P* < 0.001), and results following BiPAP were significantly greater than after O_2_ administration (*P* = 0.021). Arterial CO_2_ (PaCO_2_) results were not normally distributed and were resistant to transformation. Values were lowest in unsedated foals at T-1, and differences at this time and at T0 were significant when compared to results following administration of supplementary O_2_ and following BiPAP (all *P* < 0.001, Figure 1); differences at other times were not significant. Results following supplementary O_2_ administration were not significantly different to those obtained after BiPAP (*P* = 1.000). Hypercapnia (PaCO_2_ >60 mHg) was observed for two foals (F8 and F9) following O_2_ administration (60 and 66 mmHg, respectively), and following BiPAP on both occasions for F9 (60 and 68 mmHg). Changes to blood pH mirrored changes to PaCO_2_ and effects on lactate and blood glucose treatment attributable to treatment were not observed (Supplementary Figure S3). Blood glucose concentrations increased across all sampling times (likely due to administration of xylazine), with results at T3 (*P* = 0.013) and T5 (*P* = 0.008) significantly higher than at T0, as were results during O_2_ (*P* = 0.008) and BiPAP (*P* = 0.016, data not shown).

**Figure 2:**
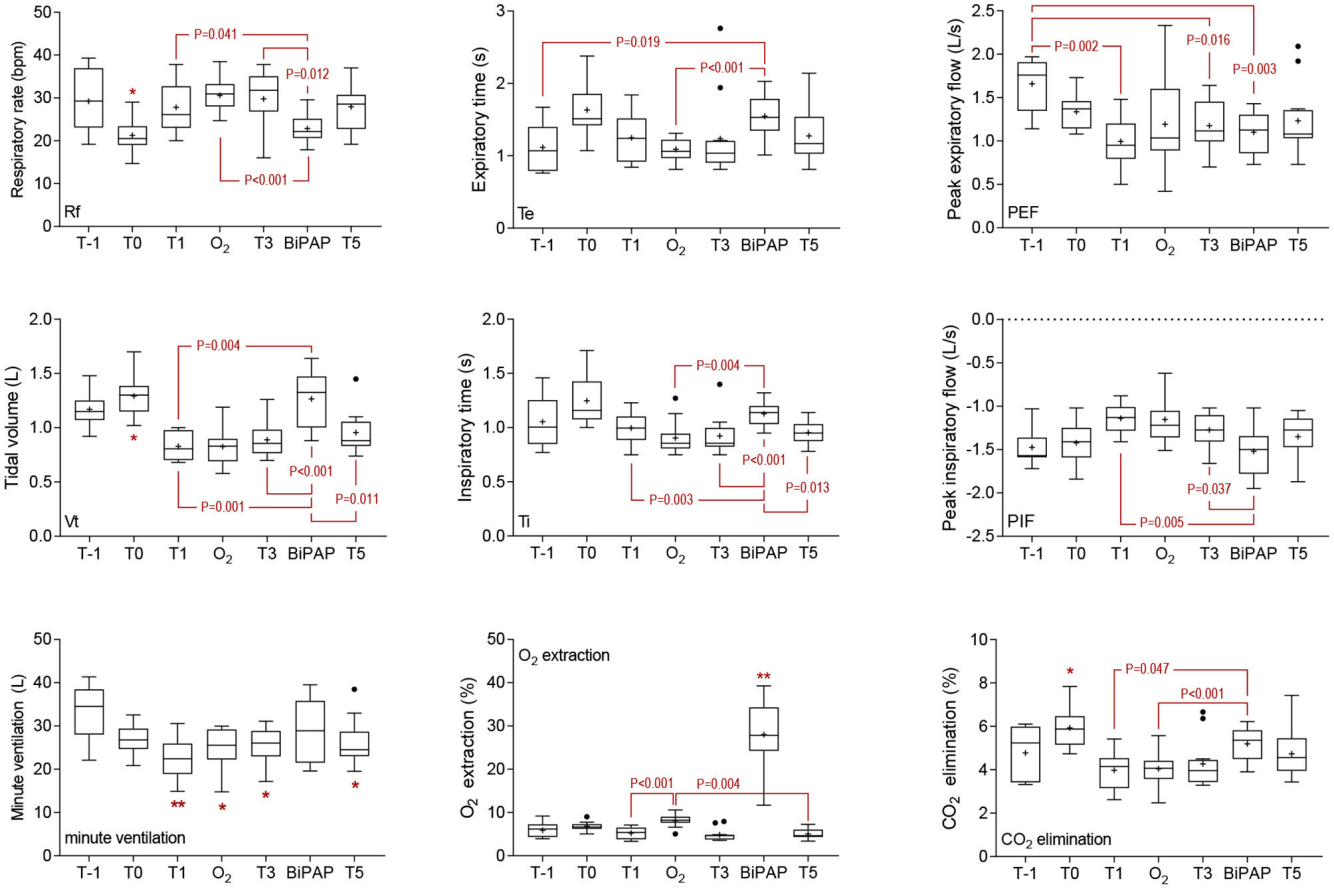
Spirometry and gas exchange results. Sedation was associated with a significant decrease in respiratory rate during spirometry (RRs) at T0, and this effect was significant (*P* < 0.05) in comparison with results at all other time points except during bi-level positive airway pressure ventilation (BiPAP). Effects on tidal volume (Vt) reciprocated those observed on RRs, with differences again observed at T0 (*P* < 0.05 when compared to other sampling points with the exception of during BiPAP). Minute ventilation was greatest in standing, unsedated foals (T-1), and significant decreases were observed at all other sampling points (^*^P < 0.05 and ^**^P < 0.01) except during BiPAP. Inspiratory (Ti) and expiratory (Te) times were longest in standing foals following sedation with diazepam (T0), but significant effects were observed only in comparison with T5 (*P* = 0.020) for Ti. For Te, comparisons between T0 and T-1 (*P* = 0.008), O_2_ (*P* = 0.006), and T5 (*P* = 0.012) were significant. Significant time effects on peak expiratory (PEF) and inspiratory (PIF) flows are shown. The administration of BiPAP was associated with greater O_2_ extraction than observed at any other time (^**^, all *P* < 0.01). Oxygen extraction was also greater during mask O_2_ administration, as shown, and at T0 (standing foals following administration of diazepam) than at T3 (*P* = 0.046) or T5 (*P* < 0.001). The elimination of CO_2_ was greatest at T0 than at any other time, except following BiPAP (^*^, all *P* < 0.05). Differences between effects observed following BiPAP administration and at other times are shown. Data are shown as mean (+), median (horizontal line) and quartiles (box), with whiskers and outliers determined by Tukey method.

Significant time-sequence interactions were observed for spirometry variables including respiratory rate during spirometry (RRs), tidal volume (Vt), inspiratory time (Ti), expiratory time (Te), and peak inspiratory flow (PIF) (Supplementary Figure S4); BiPAP was associated with significantly lower RR (*P* = 0.014) and significantly longer inspiratory (*P* < 0.001) and expiratory (*P* = 0.020) times at T2 than observed following O_2_ administration at this time. Differences at other time points were not significant, so data have been combined for analysis of treatment effects. Sedation with diazepam at T0 was associated with a significant decrease in RRs relative to each other sampling times (Figure 2), except following BiPAP (all *P* < 0.050). Respiratory rate (RRs) during BiPAP was lower than was observed at T1 (*P* = 0.041), T3 (*P* = 0.012) or following O_2_ administration (*P* < 0.001). Tidal volume (Vt) was greatest in standing foals following diazepam sedation (T0) and values observed at this time and in unsedated foals at T-1 were significantly greater than observed in recumbent foals with the exception of during BiPAP (all *P* < 0.05). The administration of BiPAP was associated with significantly greater Vt than was observed at T1 (*P* = 0.001), T3 (*P* < 0.001), T5 (*P* = 0.011) and during O_2_ administration (*P* < 0.001), and effects were more pronounced at T2 than at T4 (Supplementary Figure S4). Recumbency was associated with a significant reduction in minute ventilation relative to results from standing, unsedated foals (T-1), but this effect was not observed during BiPAP (Figure 2).

Significant effects were observed for both inspiratory and expiratory time, reflective of changes observed in RRs (Figure 2), but there was no effect on I:E ratio (data not shown). Peak inspiratory flow was greatest during BiPAP (−1.52 L/s), and significant effects were observed compared to values obtained at T1 (*P* = 0.005) and T3 (*P* = 0.037). Expiratory flows were greatest in unsedated foals (T-1), and significant differences were observed at T1 (*P* = 0.002), T3 (*P* = 0.016), and during BiPAP (*P* = 0.003).

Time and sequence effects were observed for data derived from analysis of inspired/expired gas composition due to differences following O_2_ administration or BiPAP at T2 (Supplementary Figure S5). Differences at other time points were not significant, so data have been combined for analysis of treatment effects. As expected, the administration of supplementary O_2_ was associated with an increased FiO_2_ during both mask supplementation and BiPAP, compared to FiO_2_ when breathing room air (all *P* < 0.001), and values were significantly greater during BiPAP than during administration of O_2_ only (*P* = 0.004). Oxygen concentrations in expired air were also greater following the administration of supplementary O_2_, but differences were not observed between mask O_2_ administration and BiPAP (*P* = 0.172). Oxygen extraction was much greater during BiPAP than at all other time points (all *P* < 0.005), including during O_2_ administration (*P* < 0.001, Figure 2). Oxygen extraction was also greater during mask O_2_ administration than at T1 (*P* < 0.001) or T5 (*P* = 0.004), and in standing foals at T0 relative to T3 (*P* = 0.046) and T5 (*P* < 0.001). Maximum concentrations of CO_2_ (CO_2_max) were observed at T0 in standing foals following administration of diazepam and observed differences were significant in comparison with results at T-1 (*P* = 0.016), T1 (*P* = 0.0003), T3 (*P* = 0.026), T5 (*P* = 0.0002), and following O_2_ administration (*P* = 0.031). Results after BiPAP were significantly greater than following O_2_ administration (*P* = 0.047). Minimum concentrations of CO_2_ were observed during BiPAP, and differences were significant in comparison with T3 (*P* = 0.021) and T5 (*P* = 0.013). The elimination of CO_2_ was greatest at T0 in diazepam sedated foals, with significant differences observed between results at T0 and all other sampling points, with the exception of BiPAP (all *P* < 0.030, Figure 2). Results observed following BiPAP were significantly greater than observed at T1 (*P* = 0.047) or following O_2_ administration (*P* = 0.004).

**Figure 3:**
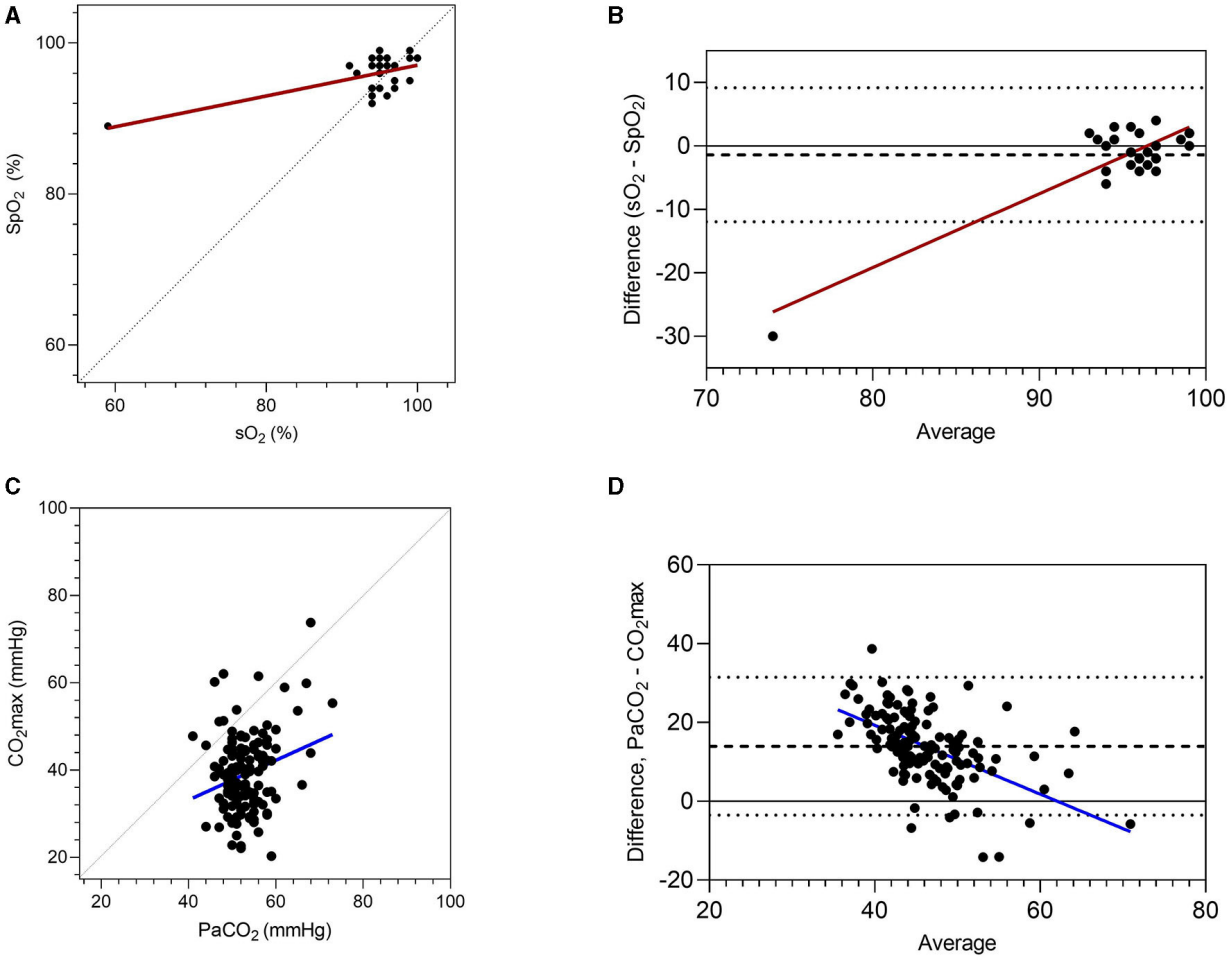
Associations between blood gas results and non-invasive measures of oxygenation (pulse oximetry, SpO_2_) and carbon dioxide accumulation (CO_2_max). Results are presented as correlations **(A,C)**, with perfect agreement (unity) shown as a dotted line. Agreement is shown following Bland-Altman analysis **(B,D)**, with the mean difference (dashed line) and limits of agreement (dotted lines) shown. Oxygenation of hemoglobin (sO_2_) and partial pressure of CO_2_ (PaCO_2_) were determined from blood gas analyses.

Paired results for pulse oximetry (SpO_2_) and hemoglobin saturation determined by blood gas analysis (sO_2_) were available for 47 data sets from the current study. SpO_2_ results correlated significantly with sO_2_ (*r* = 0.61, 95% CI 0.34–0.78, *P* < 0.001), but there was poor agreement between these two methods of assessing hemoglobin saturation (Figure 3). Although bias was minimal (1.4%, standard deviation 5.4%), the observed limits of agreement were large (−11.95 to 9.2%), and increased divergence was observed for results obtained from the most hypoxic foal. Paired results for CO_2_max and PaCO_2_ were available for 136 data sets (Figure 3). There was poor but significant correlation between results (*r* = 0.25, 95% CI 0.09–0.40, *P* = 0.003), and agreement was poor (bias 14.0 ± 8.9%) with broad limits of agreement (−3.5 to 31.5%).

## Discussion

Improved blood oxygenation was observed following BiPAP relative to mask administration of supplementary O_2_. BiPAP was also associated with decreased RR, increased Vt and increased minute ventilation, suggesting that respiratory mechanics were improved by respiratory support. Increased Vt represents a more efficient ventilation strategy than increased RR, as there is increased alveolar ventilation relative to ventilation of airway dead space, and decreased RR is likely to be associated with decreased work of breathing. Increased inspiratory pressure during BiPAP was associated with increased PIF, and the adverse effects on PEF observed during CPAP in previous studies (13) were not observed in the current study, presumably due to the lower expiratory pressures during BiPAP, relative to CPAP. As expected, increased FiO_2_ was observed during mask administration of supplementary O_2_ and during BiPAP, so observed increases in arterial oxygenation likely reflect the steeper diffusion gradient resulting from these changes. Surprisingly, FiO_2_ was higher during BiPAP than during O_2_ administration to foals. This observation was not expected during positive pressure respiratory support, where increased flow is associated with decreased partial pressure of O_2_ (27).

Increased PaCO_2_ was observed in foals following both forms of respiratory support, including hypercapnia (PaCO_2_ >60 mmHg) following O_2_ administration (two foals) or BiPAP (one foal). Hypercapnia has been reported in response to O_2_ supplementation in human neonates (28) and foals (13, 29), and may be due to reduced respiratory drive, increased metabolic rate, hypoventilation due to sedation or effects of equipment dead space. As the minimum inspired CO_2_ was greater during the administration of supplementary O_2_ than during BiPAP, it is likely that mask administration of supplementary O_2_ in the current study was associated with CO_2_ retention, a problem that might be avoided by the use of a non-rebreathing valve during BiPAP, or by nasal insufflation of oxygen. This effect was not observed during BiPAP. Despite decreased RR, minute ventilation during BiPAP was the same as observed in standing, unsedated foals in the current study suggesting that BiPAP prevented reduced ventilation associated with sedation and recumbency. However, our hypothesis, that lower expiratory pressures associated with BiPAP and improved expiratory function would ameliorate hypercapnia was not demonstrated. As was observed during CPAP (13), the observed increases in PaCO_2_ and pH in the current study were modest, and consistent with current ventilation strategies that accept increased arterial CO_2_ tension and hypercapnic acidosis (“permissive hypercapnia”) as acceptable consequences without adverse effects on outcome (30, 31), and with possible therapeutic effects (32). Sedation, and the supraphysiological PaO_2_ values observed in the current study, might have contributed to the observed hypercapnia.

Although reversed within 10 min of cessation of BiPAP, the observed hypercapnia demonstrates the necessity for close monitoring during the implementation of respiratory support in equine neonates. Evaluation of NIV should consider effects on both oxygenation and carbon dioxide, but the current study demonstrated poor agreement between non-invasive methods of continuous monitoring of O_2_ saturation and CO_2_ accumulation. Pulse oximetry has been recommended as an appropriate alternative to invasive sampling for determination of hemoglobin saturation, and previous studies have suggested that placement of transmission or reflectance sensors on the lip or tongue ensures the most reliable assessment of SaO_2_ in foals (33). However, bias and limits of agreement in that study (33) were similar to those observed in the current study and well-outside accepted standards of care (34). Probe placement on the lip or tongue was not possible during mask administration of respiratory support in the current study, and was not tolerated by unsedated foals. Direct measurement of PaO_2_ and sO_2_ by co-oximetry is more accurate than blood gas analysis for determination of oxygenation (35), but both techniques require arterial samples, and neither provides an immediate result or allows for continuous monitoring. Arterial samples can be difficult to obtain in hypovolaemic foals or animals with distal limb oedema, and risks associated with arterial puncture include pain, hemorrhage, arterial injury, aneurism formation, thrombosis, and distal ischaemia. End-tidal CO_2_ (PETCO_2_) is commonly monitored during anesthesia as an indirect measure of PaCO_2_. Whilst PETCO_2_ has been reported as an acceptable technique for monitoring of neonatal foals (34), studies during NIV in people have suggested that, as observed in the current study, the technique was not predictive of PaCO_2_ or changes in PaCO_2_ (36). The association between PETCO_2_ and PaCO_2_ assumes that the patient exhales fully, and that end-expiratory gases approximate gas composition in the alveoli. This assumption is not valid if, as we have hypothesized, foals are not exhaling completely. For this reason peak expired CO_2_ concentrations have been termed FeCO_2_max in the current study. Alternative techniques to assess ventilatory function, such as volume capnography and electrical impedance tomography (37), offer greater capacity to more accurately assess response to respiratory support.

A number of limitations were noted in the current study. Findings in healthy foals with pharmacologically-induced respiratory insufficiency may not be predictive of responses in neonates with spontaneous disease, and characterization of the technique in neonates with spontaneous respiratory disease is required. We were unable to document alveolar ventilation, physiological dead space or PEEPi in the current study, which was designed to evaluate a readily available intervention and monitoring strategies that might be readily implemented in a practice setting. The assessment of non-invasive monitoring was based on a small number of observations, and the data set did not include results from foals with severe hypoxaemia or hypercapnia.

## Conclusions

Consistent with previous studies evaluating CPAP, BiPAP was an effective respiratory support strategy for healthy foals with pharmacologically induced respiratory insufficiency. BiPAP was associated with increased PaO_2_, more efficient gas exchange and changes in respiratory mechanics including increased tidal volume, decreased respiratory rate, and increased peak inspiratory flow. The technique preserved minute ventilation in the face of reduced ventilation observed at other times associated with sedation and recumbency, but was associated with modest increase in PaCO_2_. As in previous studies, the use of a commercially available ventilator intended for at-home care of adults with chronic obstructive respiratory conditions or sleep apnoea represents an available and potentially cost effective option for use in equine practice, although there is a need for careful and frequent monitoring of patient oxygenation and ventilation (carbon dioxide elimination) during NIV. Our results suggest that monitoring of alveolar ventilation, pressure-volume curves, and PEEPi might be important for effective NIV of foals and to better characterize the response of foals to respiratory support. The use of lower expiratory pressures in the current study did not prevent hypercapnia, and increases in PaCO_2_ observed in the current study were similar to those observed previously during CPAP in healthy foals. Observed effects on PaCO_2_ were rapidly reversed and predominantly within acceptable bounds for permissive hypercapnia. Although not a primary objective of the current study, our results suggested the non-invasive monitoring approaches used in this study were not reliable, and techniques are needed for more accurate, non-invasive assessment of respiratory function in foals during NIV.

## Data Availability Statement

The raw data supporting the conclusions of this article will be made available by the authors, without undue reservation.

## Ethics Statement

The animal study was reviewed and approved by Charles Sturt University Animal Care and Ethics Committee (ACEC A18044).

## Author Contributions

All authors contributed to experimental design and conduct of experimental procedures. SR and LB contributed to data analysis and early manuscript drafts, with editing and correction by all authors.

## Funding

This study was funded by AgriFutures Australia (PRJ-011159). A contribution towards costs of publication was received from Charles Sturt University's Tri-Faculty Open Access Publication Scheme.

## Conflict of Interest

The authors declare that the research was conducted in the absence of any commercial or financial relationships that could be construed as a potential conflict of interest.

## Publisher's Note

All claims expressed in this article are solely those of the authors and do not necessarily represent those of their affiliated organizations, or those of the publisher, the editors and the reviewers. Any product that may be evaluated in this article, or claim that may be made by its manufacturer, is not guaranteed or endorsed by the publisher.
