# Supplementary material for: Bi-Level Positive Airway Pressure for Non-invasive Respiratory Support of Foals
Source: Front Vet Sci. 2021 Sep 29;8:741720. doi: 10.3389/fvets.2021.741720 (PMC8511517; doi:10.3389/fvets.2021.741720)

Supplementary Table S1: Research foals and treatment schedule used in the current study. All foals were sired by a Connemara stallion out of Standardbred (F2 and F7) or Thoroughbred (F5, F6, F8, F9) mares. There were two colts (F2 and F6) and four fillies. DOB, date of birth; BW, body weight; T2, treatment allocated to T2; T4, treatment allocated to T4; interval, time between Phase 1 and Phase 2 for each foal.

| | | | Phase 1 | | | | | Phase 2 | | | | | |
|---|---|---|---|---|---|---|---|---|---|---|---|---|---|
| Foal | Gender | DOB | Date | Age (d) | BW (kg) | T2 | T4 | Date | Age (d) | BW (kg) | T2 | T4 | Interval (d) |
| F2 | M | 27/08/2018 | 18/10/2018 | 52 | 111 | $O_2$ | BiPAP | 24/10/2018 | 58 | 126 | BiPAP | $O_2$ | 6 |
| F6 | M | 31/08/2018 | 19/10/2018 | 49 | 121 | BiPAP | $O_2$ | 24/10/2018 | 54 | 131 | $O_2$ | BiPAP | 5 |
| F9 | F | 1/09/2018 | 19/10/2018 | 48 | 125 | $O_2$ | BiPAP | 23/10/2018 | 52 | 132 | BiPAP | $O_2$ | 4 |
| F7 | F | 5/09/2018 | 22/10/2018 | 47 | 110 | BiPAP | $O_2$ | 26/10/2018 | 51 | 114 | $O_2$ | BiPAP | 4 |
| F8 | F | 9/09/2018 | 23/10/2018 | 44 | 114 | $O_2$ | BiPAP | 26/10/2018 | 47 | 118 | BiPAP | $O_2$ | 3 |
| F5 | F | 28/10/2018 | 13/12/2018 | 46 | 86 | BiPAP | $O_2$ | 18/12/2018 | 51 | 92 | $O_2$ | BiPAP | 5 |

Supplementary Figure S1: BiPAP ventilator, spirometer and gas analyser (top), mask with vented non-rebreathing elbow valve (A) and spirometry flow head (B). Oxygen was added to the circuit at C.

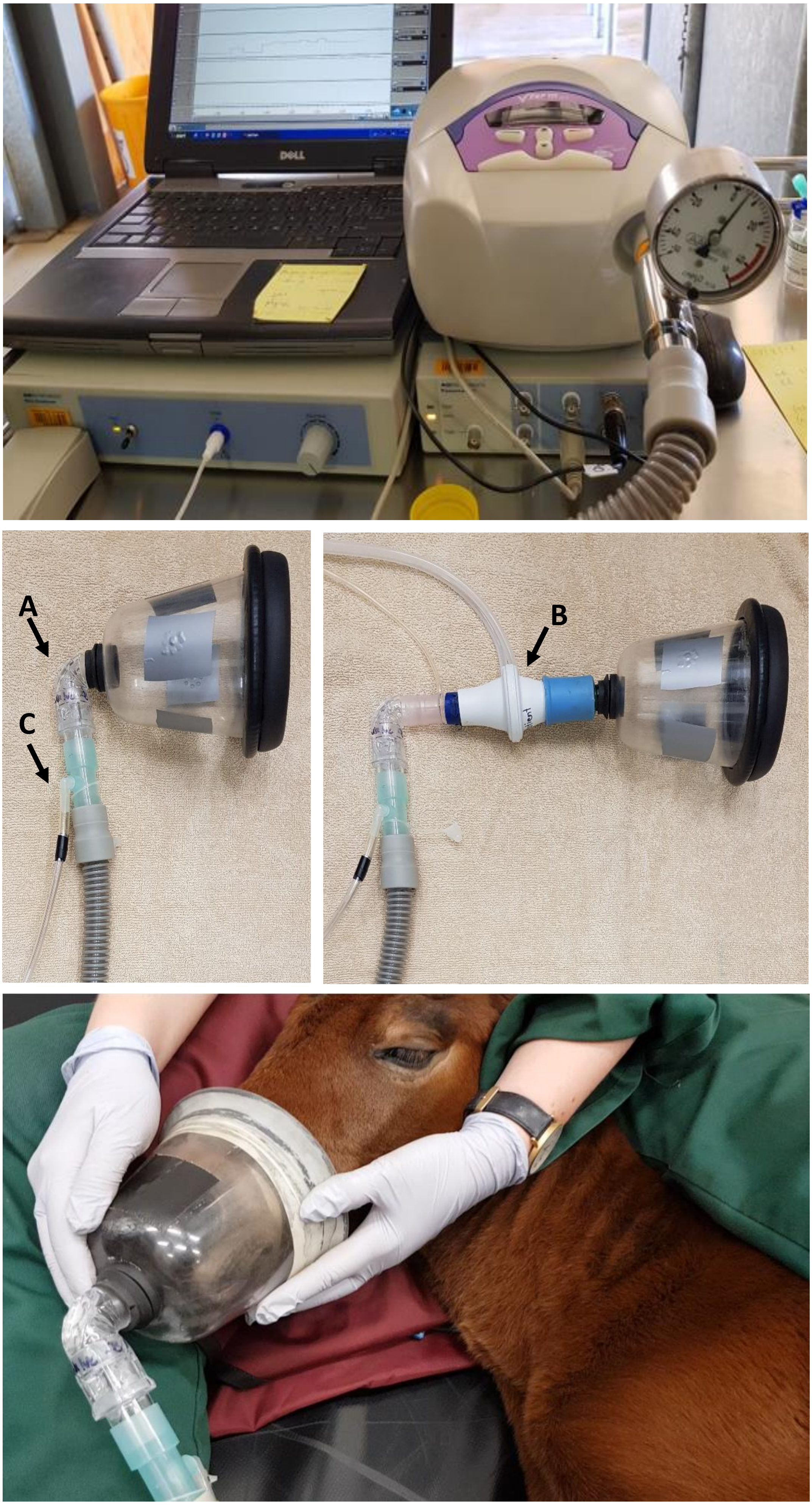

Supplementary Figure S2: Effect of sequence (BiPAP or supplementary $O_2$ as the first administered treatment) on heart rate (HR), observed respiratory rate (RR), rectal temperature (temp) and mean arterial pressure (MAP). A significant effect of time ($P<0.001$) was observed for all variables except MAP ($P=0.529$), but there were no effects attributable to sequence ($O_2$–BiPAP, supplementary $O_2$ at T2, BiPAP at T4; BiPAP-$O_2$, BiPAP at T2, supplementary $O_2$ at T4). Data are shown as mean and standard deviation.

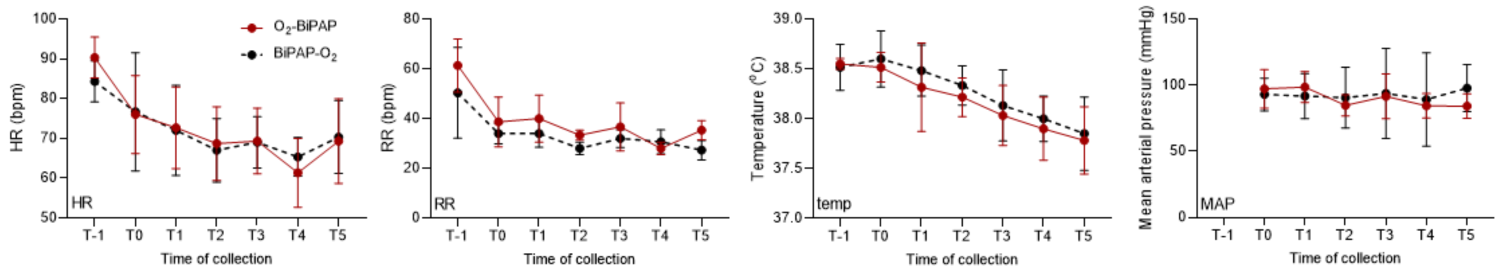


Supplementary Figure S3: Effect of sequence (BiPAP or supplementary $O_2$ as the first administered treatment) on blood gas parameters. A significant effect of time was observed for the partial pressure of $O_2$ ($PaO_2$), $CO_2$ ($PaCO_2$) and pH (all $P<0.001$), and for lactate ($P=0.012$). There were no effects attributable to sequence ($O_2$–BiPAP, supplementary $O_2$ at T2, BiPAP at T4; BiPAP-$O_2$, BiPAP at T2, supplementary $O_2$ at T4). Data are shown as mean and standard deviation.

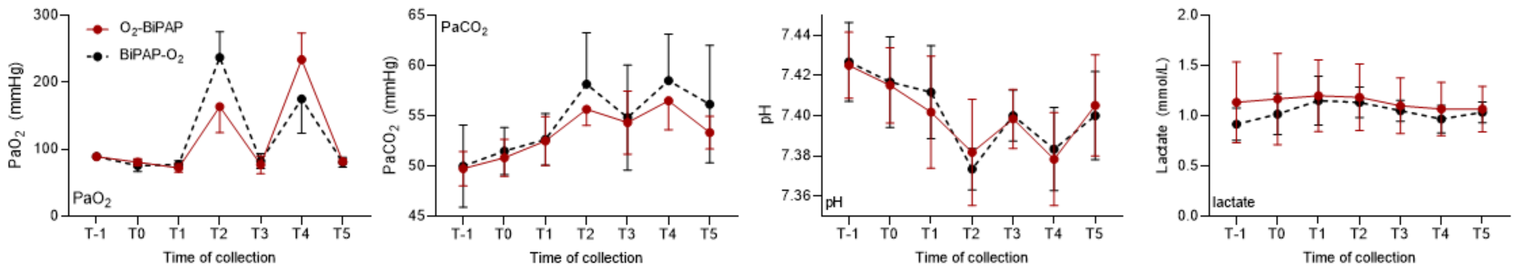

Supplementary Figure S4: Effect of sequence (BiPAP or supplementary $O_2$ as the first administered treatment) on spirometry results. A significant effect of time was observed for respiratory rate during spirometry (RR, P=0.032), tidal volume (Vt, P<0.001), inspiratory time (Ti, P=0.027), expiratory time (Te, P=0.05), peak expiratory flow (PEF, P<0.001) and peak inspiratory flow (PIF, P=0.039). Significant time-sequence interactions were observed for RRs (P<0.001), Vt (P<0.001), Ti (P=0.008), Te (P<0.001) and PIF (P=0.001) with significant effects observed between results obtained following $O_2$ supplementation and following BiPAP at T2, as shown; other pairwise comparisons were not significant. The time-sequence interaction for PEF was not significant (P=0.291). Data are shown as mean and standard deviation.

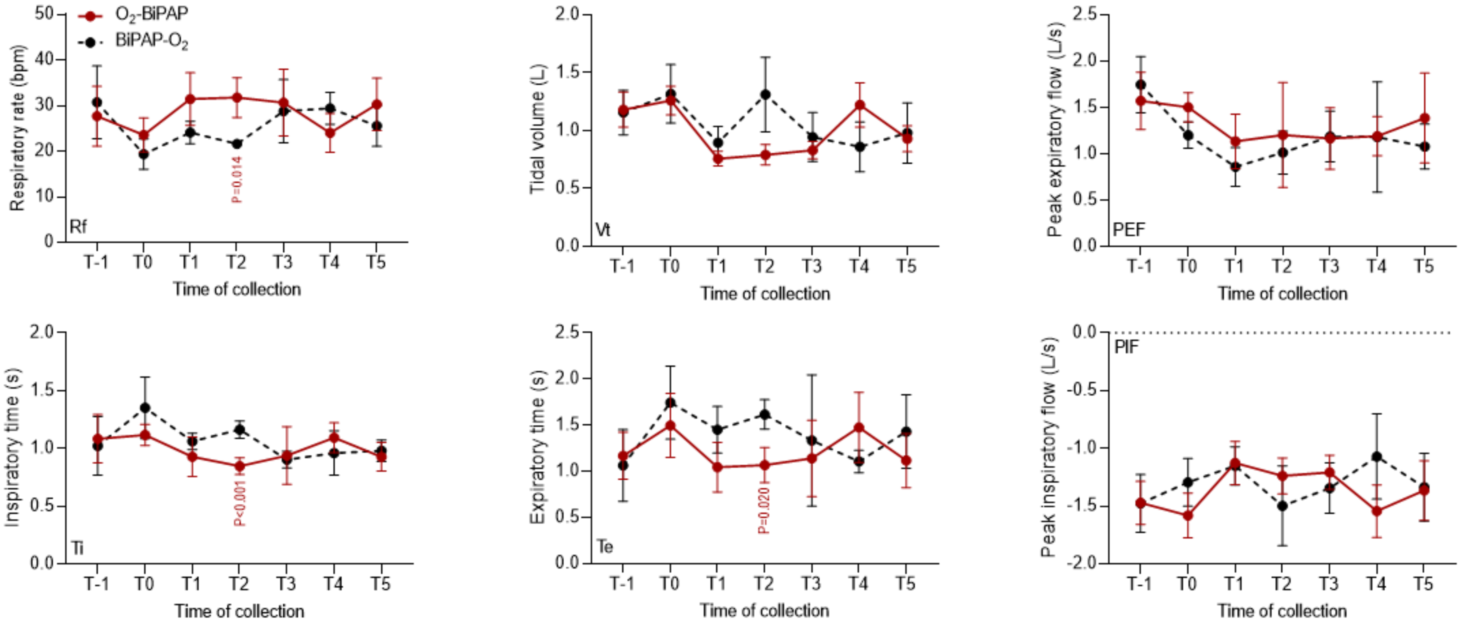

Supplementary Figure S5: Effect of sequence (BiPAP or supplementary $O_2$ as the first administered treatment) on inhaled and exhaled gas parameters. Significant time effects were observed for the maximum inspired O2 concentration (FiO2max), minimum observed $O_2$ concentration ($FiO_2$min) and $O_2$ extraction (all $P<0.001$). Significant time-sequence interactions were observed (all $P\leq0.001$), and significant pairwise comparisons are shown. Significant time effects were also observed for maximum expired $CO_2$ concentrations ($FeCO_2$max, P=0.023), $CO_2$ elimination (P=0.001), but not for minimum $CO_2$ concentrations (insp $CO_2$, P=0.438). Time-sequence interactions were significant for all $CO_2$ parameters (P=0.014, P=0.002 and P=0.004, respectively), but pairwise comparisons were not significant. There were no effects attributable to sequence. Data are shown as mean and standard deviation.

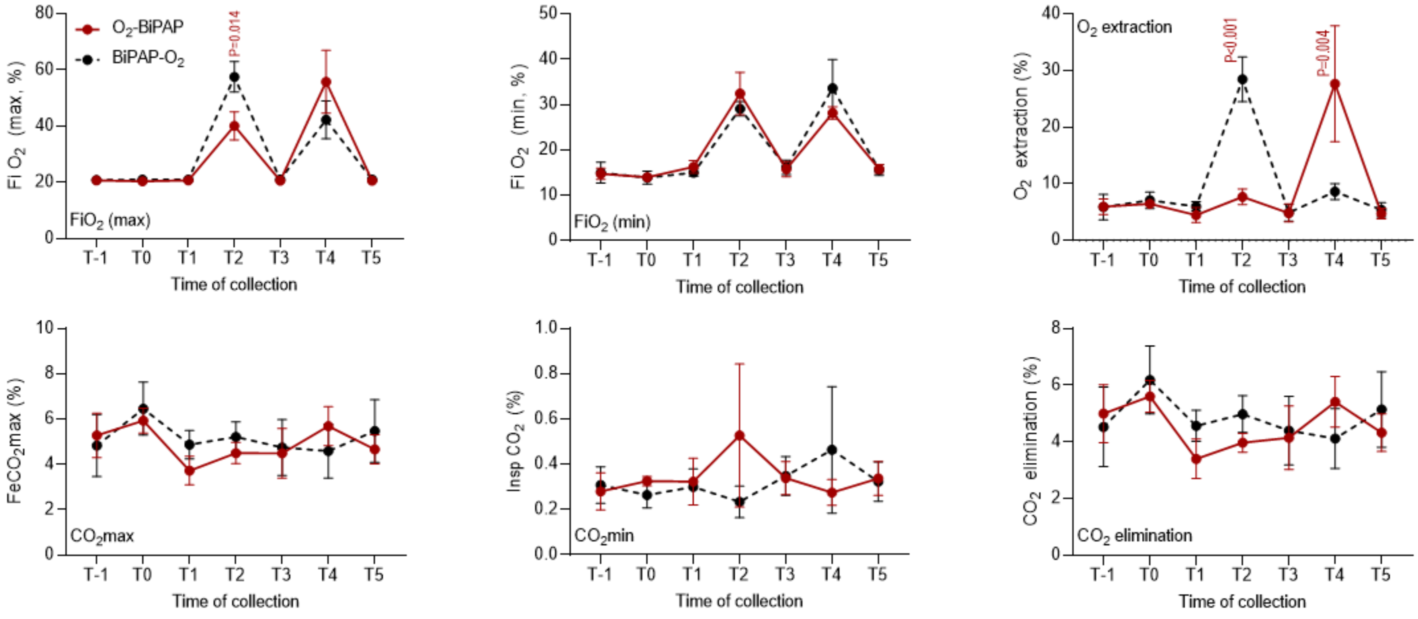

Supplement: Supplementary file 1 [file Presentation_1.PDF]
